# The Influence of Post-Weld Heat Treatment on the Microstructure and Fatigue Properties of Sc-Modified AA2519 Friction Stir-Welded Joint

**DOI:** 10.3390/ma12040583

**Published:** 2019-02-15

**Authors:** Robert Kosturek, Lucjan Śnieżek, Marcin Wachowski, Janusz Torzewski

**Affiliations:** Faculty of Mechanical Engineering, Military University of Technology, 2 gen. W. Urbanowicza str., 00-908 Warsaw, Poland; lucjan.sniezek@wat.edu.pl (L.Ś.); marcin.wachowski@wat.edu.pl (M.W.); janusz.torzewski@wat.edu.pl (J.T.)

**Keywords:** friction stir welding, heat treatment, AA2519, microstructure, fatigue, fractography

## Abstract

The aim of this research was to investigate the influence of post-weld heat treatment (PWHT, precipitation hardening) on the microstructure and fatigue properties of an AA2519 joint obtained in a friction stir-welding process. The welding process was performed with three sets of parameters. One part of the obtained joints was investigated in the as-welded state and the second part of joints was subjected to the post-weld heat treatment (precipitation hardening) and then investigated. In order to establish the influence of the heat treatment on the microstructure of obtained joints both light and scanning electron microscopy observations were performed. Additionally, microhardness analysis for each sample was carried out. Fatigue properties of the samples in the as-welded state and the samples after post-weld heat treatment were established in a low-cycle fatigue test with constant true strain amplitude equal to ε = 0.25% and cycle asymmetry coefficient R = 0.1. Hysteresis loops together with changes of stress and plastic strain versus number of cycles are presented in this paper. The fatigue fracture in tested samples was analyzed with the use of scanning electron microscope. Our results show that post-weld heat treatment of AA2519 friction stir-welded joints significantly decreases their fatigue life.

## 1. Introduction

High-strength aluminum alloys are very interesting engineering materials due to their high specific strength, forming abilities, good mechanical properties at low temperature and corrosion resistance [1,2,3]. Some of these alloys also present good ballistic resistance, which makes them especially attractive to the military, as well as, the space industry [1,4]. AA2519 is a heat treatable aluminum-copper alloy with copper content within the 5.3–6.4% range, used in the military for advanced amphibious assault vehicles (AAAV) [5]. The precipitation hardening of this alloy is realized by a two-step heat treatment—the solution treatment (annealing in 530 °C/2 h and cooling in cold water) and artificial aging (165 °C/10 h) [6,7,8]. After this process AA2519 alloy is strengthened by θ′ precipitates, semi-coherent metastable Al_2_Cu phase with body-centered tetragonal crystal structure, which increases its mechanical properties significantly [7]. Additionally the modification of AA2519 alloy used in this research consists of the addition of scandium and has been developed by the Institute of Non-Ferrous Metals, Light Metals Division in Skawina (Poland). Adding scandium to aluminum alloys affects their properties significantly by increasing their mechanical properties, recrystallization temperature and causes grain refinement due to presence of Al_3_Sc precipitates [9,10,11,12]. Despite the advantages of AA2519 alloy, the high concentration of copper causes problems with its welding, especially hot cracking during solidification of the weld [13,14]. One of the most promising technologies for joining aluminum alloys (including AA2519) is friction stir-welding [15,16,17,18,19,20,21,22]. This solid-state welding process is based on friction between workpieces and the rotating tool, which generates heat leading to plasticizing of the material to be welded [15,16,17]. The movement of the rotating tool along the edges of two workpieces causes a mixing of the plasticized material, and as a result the creation of a joint between them [15,17,21]. The process of welding significantly influences the microstructure of joined alloy especially dissolution and coarsening of strengthening precipitates which causes a decrease of joint mechanical properties compared to the base material [21,22,23,24]. An approach worth considering to avoid this disadvantageous change in distribution of strengthening precipitates is to join the alloy in a non-strengthened state and then subject it to the post-welded heat treatment (PWHT) [15,23,24,25]. Most of the research concerned with friction stir welding of high-strength aluminum alloys is focused on basic mechanical properties of the joint established in tensile tests, omitting investigation of fatigue properties which are far more important in terms of application for construction of machines—particularly in the aerospace industry [2,18,19,21,22]. It has been reported that PWHT can increase the mechanical properties of high-strength aluminum alloys established during tensile tests, such as tensile strength and elongation [15,24]. Although the increase of joint efficiency of heat-treated welds is significant, the response of such a joint on the cyclic load is rarely the subject of investigations. The literature does not contain a research concerned with the influence of post-weld heat treatment of an AA2519 friction stir-welded joint on its fatigue properties. The present work is aimed at investigating the microstructure of AA2519 friction stir welded (FSW) joints in the as-welded state and after PWHT and to explore its influence on the low-cycle fatigue properties of welds.

## 2. Materials and Methods

The workpieces to be joined were 5 mm thick sheets made of AA2519-O alloy with chemical composition as presented in Table 1.

The friction stir welding process has been performed by using ESAB FSW Legio 4UT machine (Military University of Technology, Warsaw, Poland) with the set of welding parameters presented in Table 2.

**Figure 1:**
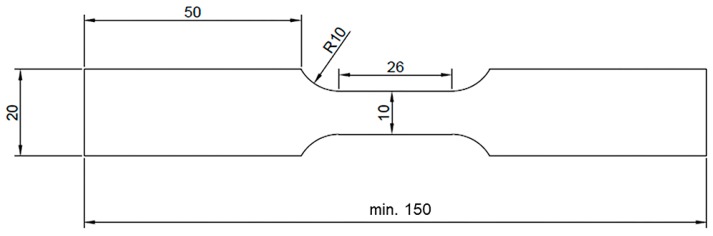
Scheme of sample for fatigue testing. All dimension are in mm.

For each welding process axial force was equal to 17 kN and the tilt angle of Triflute type tool was set to 2°. From the welded workpiece there were cut two types of samples, designated as HT0 and HT1. The HT0 samples were investigated in the as-welded state, and HT1 samples were subjected to the post-welded precipitation hardening process and then investigated. The precipitation hardening process has been performed by solution treatment in 530 °C for 2 h and cooling in cold water, and then aging in 165 °C for 10 h. The welded joints were sectioned perpendicular to the welding direction where metallurgical examinations and hardness measurements were carried out. The very important matter in the friction stir welding process is that the material flow around the tool is not symmetrical. A friction stir-welded joint has its advancing side (AS, where the direction of the tool rotation is accordant with the welding direction) and retreating side (RS, where the direction of the tool rotation is opposed to the welding direction). In this study every cross-section of the joint subjected to the microstructure observations or microhardness analysis has a retreating side on its right side. In order to investigate the joint microstructure, the samples were examined using a digital light microscope (Olympus LEXT OLS 4100, Military University of Technology, Warsaw) and scanning electron microscope (Jeol JSM-6610, Military University of Technology, Warsaw,) with energy-dispersive x-ray spectroscopy (EDX) detector. The samples were etched by using Kroll reagent (20 mL H_2_O + 5 mL HNO_3_ + 3 drops of HF) with etching time equal to 15 s. The Vickers microhardness measurements of the polished cross sections were performed across the welds by applying a load of 0.1 kg. The top left corner of each sample has been set as “point zero” for the measurements. For each joint, microhardness distributions were prepared for the upper, middle and lower part of the cross section: 0.7 mm, 2.8 mm and 4.2 mm from the top respectively. Fatigue properties were established on an Instron 8802 Servohydraulic Fatigue Testing System (Military University of Technology, Warsaw, Poland) with constant true strain amplitude ε = 0.25%, cycle asymmetry coefficient R = 0.1 and frequency equal to f = 1 Hz. The strain during testing has been measured using a 2520–603 dynamic extensometer. The scheme of sample for fatigue testing is presented in Figure 1.

## 3. Results

**Figure 2:**
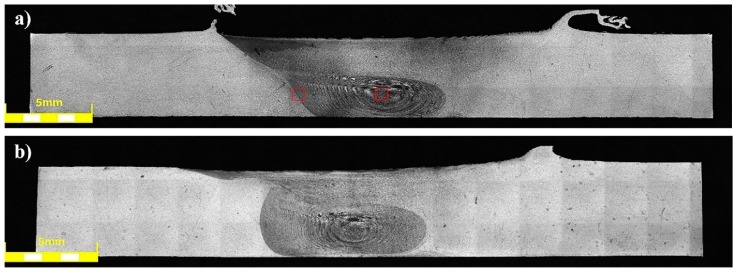

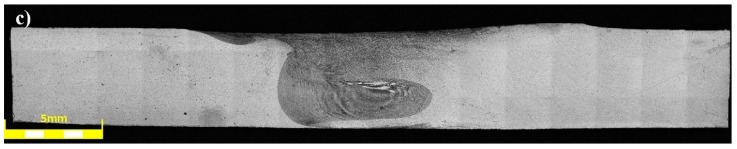
Light microscopy image of friction stir welded (FSW) joints macrostructure in the as-welded state: (**a**) 400-100-HT0, (**b**) 800-100-HT0, (**c**) 800-200-HT0.

The joints obtained in the as-welded state (HT0 samples) have a typical for friction stir-welding process macrostructure consisting of a stir zone localized in the center of the joint, a thermo-mechanically affected zone, and a heat affected zone (Figure 2).

**Figure 3:**
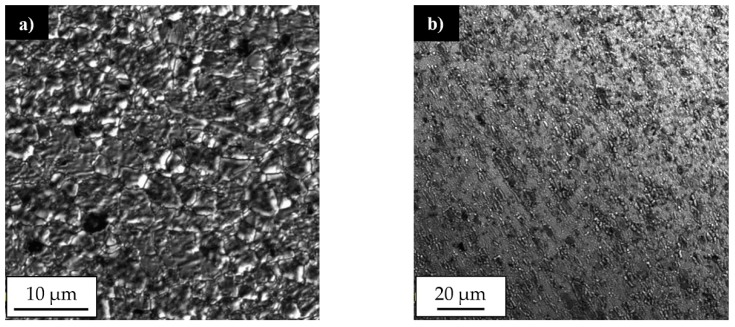
Light microscopy image of 400-100-HT0 sample microstructure in: (**a**) stir zone, (**b**) boundary between stir zone and thermo-mechanically affected zone. (Red marked areas in Figure 2a).

Our results showed that the investigated joints are free of any imperfections, such as voids, cracks, kissing bound or tunneling effect. The shape of the stir zone for each sample differed due to different parameters of welding process. In case of joints welded with a tool rotation speed equal to 800 rpm it is possible to observe a much rounded shape on the advancing side of the stir zone compared to the angular shape of this area in 400-100-HT0 sample. The investigation on the macrostructure of joints 800-100-HT0 and 800-200-HT0 allowed us to conclude that size of the stir zone is closely related to the welding velocity. Sample 800-200-HT0 had a significant smaller stir zone than in the case of a joint which has been welded with a twofold lower welding velocity. The most noticeable differences concern the retreating side of the joint, as well as, the lower part of the joint. The light microscopy observations of the stir zone of 400-100-HT0 sample revealed the microstructure characterized by the presence of fine, dynamically recrystallized grains with their size about 5 μm (Figure 3a). The boundary between stir zone and thermo-mechanically affected zone in this sample was the subject of further investigation and its light microscopy image is presented in Figure 3b.

**Figure 4:**
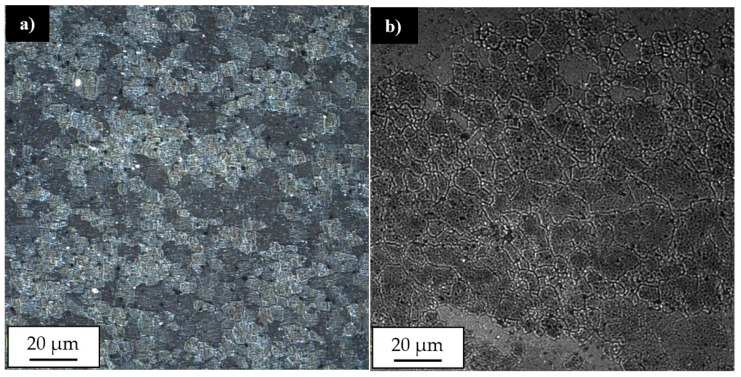
Light microscopy image of the stir zone microstructure in: (**a**) 800-100-HT0 sample, (**b**) 800-200-HT0 sample.

The light microscopy observations of this zone allowed us to observe a very specific boundary between deformed, elongated grains of thermo-mechanically affected zone and equiaxial, fine grains of the stir zone formed due to a dynamic recrystallization process. The stir zone microstructures of the joints obtained with tool rotation speed equal to 800 rpm are presented in Figure 4a,b.

**Figure 5:**
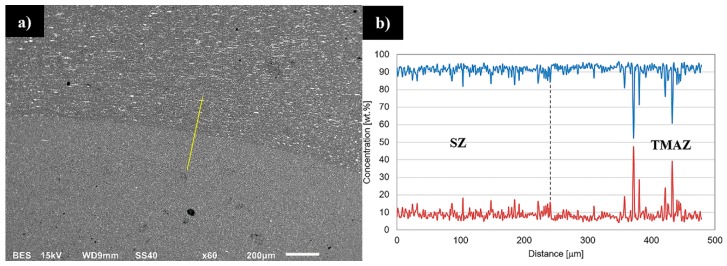
Scanning electron microscopy image of the boundary between stir zone and thermo-mechanically affected zone in 800-100-HT sample (**a**) with linear analysis of the chemical composition; (**b**) (yellow marker). Lines designation: Al (blue), Cu (red).

Although the stir zones were formed during friction stir welding due to dynamic recrystallization process it is possible to observe the differences between their grainy microstructures. In case of joint in 800-100-HT0 sample the microstructure of stir zone is more homogeneous than in 800-200-HT0 and its grains size about 5 μm is comparable to 400-100-HT0 sample. The stir zone microstructure of 800-200-HT0 is far more heterogeneous and it is possible to observe grains of 2–3 μm and the far larger ones with their size about 15–20 μm. Additionally, the scanning electron microscopy observations revealed the differences in concentration of the alloying elements between the thermo-mechanically affected zone and the stir zone. This phenomenon is most noticeable in case of 800-100-HT0 sample which has the highest ratio of tool rotation speed to tool traverse speed which results in the longest time affecting the workpiece material by the rotating tool (Figure 5a). The brighter image of the stir zone suggests a higher concentration of the elements heavier than aluminum in this area. It is also possible to observe lower participation of the large Al_2_Cu precipitates in the stir zone compared to the thermo-mechanically affected zone. This observation allowed us to draw a conclusion that Al_2_Cu precipitates dissolve in the stir zone during the friction stir-welding process. The results of the linear analysis of the chemical composition indicates decreasing participation of the aluminum in the stir zone (Figure 5b). At the same time, the fluctuations of aluminum and copper concentrations occurring due to the presence of Al_2_Cu precipitates disappears.

**Figure 6:**
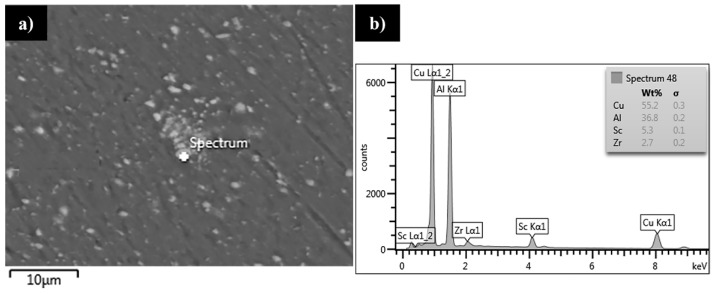
Scanning electron microscopy image of the precipitates in the stir zone (**a**), EDX area analysis of the chemical composition of the precipitate (**b**).

The scanning electron microscopy observations of the stir zone in the 800-100-HT0 sample allowed us to observe a fine dispersion of precipitates with its size about 1 μm, which have not been dissolved due to friction of the stir-welding process in this area (Figure 6a).

**Figure 7:**
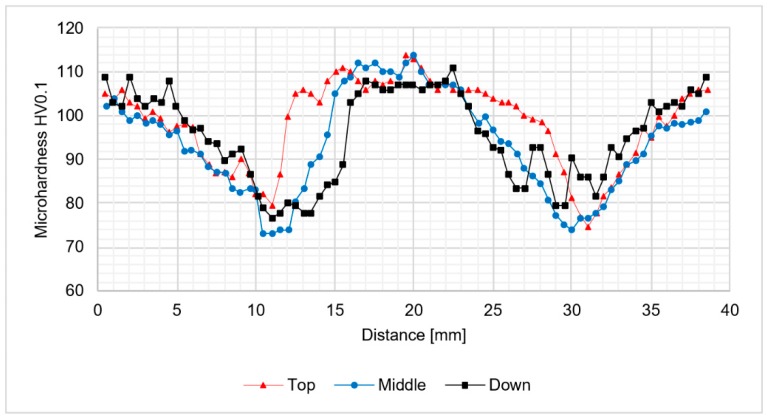
The results of 400-100-HT0 joint microhardness analysis.

**Figure 8:**
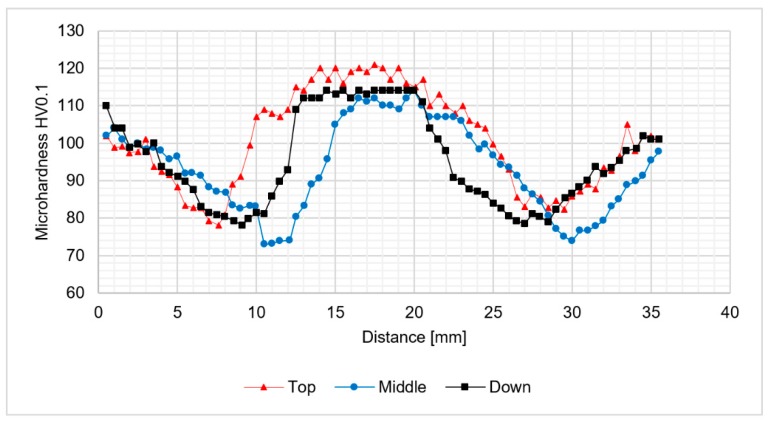
The results of 800-100-HT0 joint microhardness analysis.

**Figure 9:**
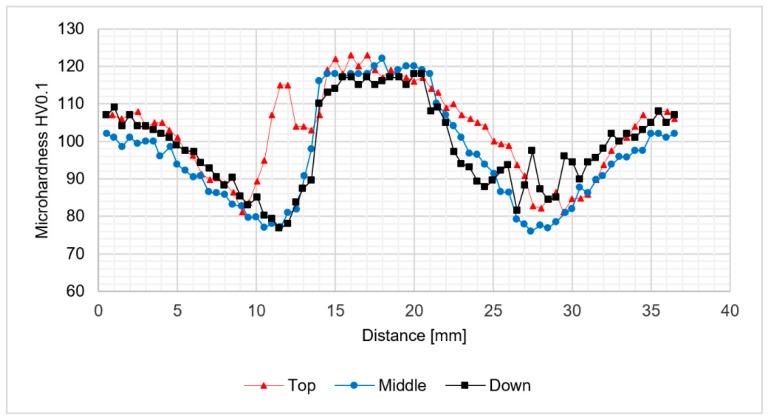
The results of 800-200-HT0 joint microhardness analysis.

In order to establish the chemical composition of the precipitates occurring in the stir zone the EDX area analysis of the chemical composition was performed (Figure 6b). The results indicate a high concentration of zirconium and scandium in the precipitates which were not dissolved in the stir zone. It suggests the low solubility during severe plastic deformation in the elevated temperature of the precipitates rich in scandium and zirconium. The results of microhardness analysis of the samples in the as-welded state (HT0) are presented at Figure 7, Figure 8 and Figure 9.

**Figure 10:**
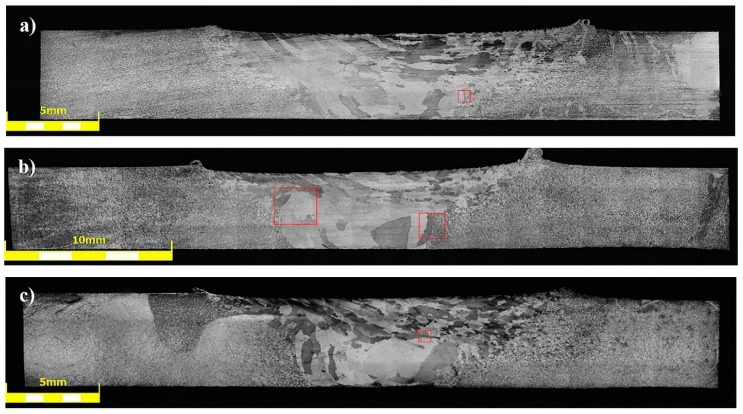
Light microscopy image of macrostructure of FSW joints subjected to the post-weld heat treatment: (**a**) 400-100-HT1, (**b**) 800-100-HT1, (**c**) 800-200-HT1.

The analysis of microhardness distribution at the cross-section of the joints allowed us to notice the increase of microhardness in the stir zone due to the occurence of fine, dynamically recrystallized grains in this area. The most significant increase was observed for the samples obtained using 800 rpm tool rotation speed (Figure 8 and Figure 9). In case of 400-100-HT0 sample the microhardness of the stir zone is about 5-10 HV0.1 higher compared to the microhardness of the base material (Figure 7). At the same time, the increase of the microhardness in the stir zone for 800-100-HT0 and 800-200-HT0 samples was estimated to value 10-20 HV0.1 (Figure 8 and Figure 9). The low hardness zone was localized at the boundary between thermo-mechanically affected zone and heat-affected zone and for each sample the lowest value in this area was noticed for the middle path of the measurements. The formation of this specific zone was related to the fact that in this area occurs the highest ratio of the heat input to the plastic deformation, which results in grain growth which is not compensated for by strain hardening. The lowest values of the hardness in the low hardness zone have been registered for 400-100-HT0 and 800-100-HT0 samples and are equal to 73 HV0.1 and 73.9 HV0.1 respectively (Figure 7 and Figure 8). In the case of the 800-200-HT0 sample the lowest hardness is equal to 76 HV0.1 (Figure 9). The macrostructures of the joints subjected to the post-weld heat treatment (precipitation hardening) are presented at Figure 10.

**Figure 11:**
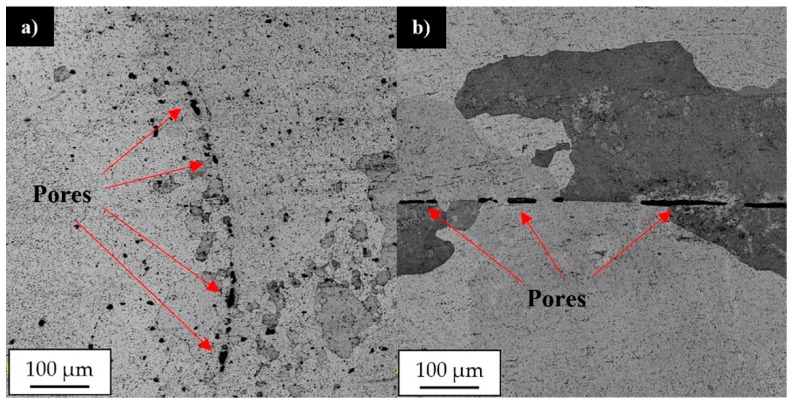
Light microscopy image of pores in the microstructure of: (**a**) 400-100-HT1 sample, (**b**) 800-200-HT1 sample. (Red marked areas in Figure 10a,c).

The post-weld heat treatment changed the macrostructure of the joints significantly. Our results show that in the stir zone and the upper part of the thermo-mechanically affected zone the grainy microstructure suffered abnormal grain growth. This phenomenon occurred in each investigated sample, despite the welding parameters used. The grains in the stir zone undergo the greatest overgrowth and as a result their size has changed from the initial value in the as-welded state of 5 μm to the size even of 1–2 mm. The very specific structure of the grains in the upper part of the thermo-mechanically affected zone seemed to maintain texture which was formed due to the friction stir-welding process. The second important result revealed during light microscopy observations is the presence of the pores in the microstructure of joints subjected to the post-weld heat treatment (Figure 11).

**Figure 12:**
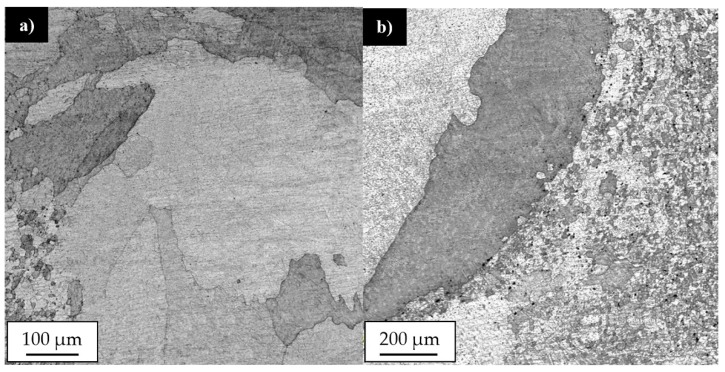
Light microscopy image of 800-100-HT1 sample microstructure in boundary between stir zone and thermo-mechanically affected zone at: (**a**) advancing side, (**b**) retreating side of the joint.

The pores were mainly localized at the boundary line between the stir zone and thermo-mechanically affected zone of the samples in the as-welded state. The pores have not been found in the base material after heat treatment. The sample 800-100-HT1 is characterized by occurrence of the smallest pores (Figure 12).

**Figure 13:**
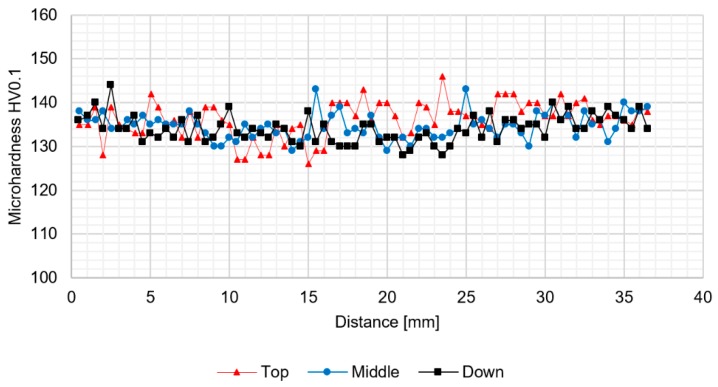
The results of 400-100-HT1 joint microhardness analysis.

**Figure 14:**
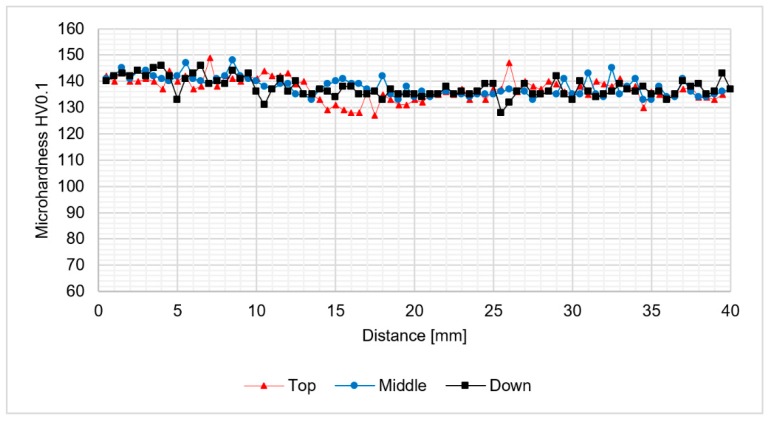
The results of 800-100-HT1 joint microhardness analysis.

**Figure 15:**
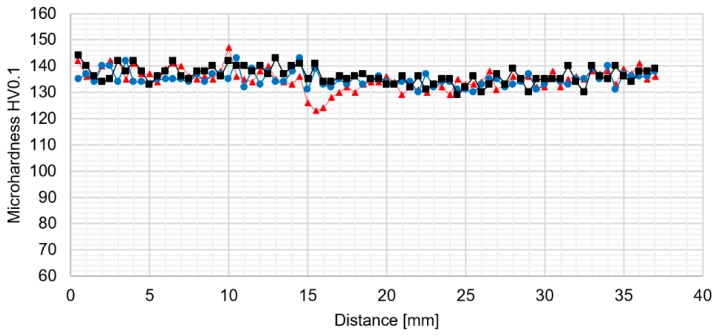
The results of 800-200-HT1 joint microhardness analysis.

The light microscopy observations of 800-100-HT1 sample microstructure allowed us to observe a very specific boundary between zone consisting of fine grains with size about 5–10 μm and the large grain size of 2 mm (Figure 12b). Additionally, this area consists small pores. A similar boundary occurs in all investigated samples and it is possible to observe it at macrostructure images of the joints (Figure 10a–c). The results of microhardness analysis of the samples which have been subjected to the post-weld heat treatment (HT1) are presented in Figure 13, Figure 14 and Figure 15.

The results of the microhardness distribution on the cross-section of the samples subjected to the post weld heat treatment indicate uniform hardening of AA2519 alloy. Our results show that the small fluctuations in microhardness occured in the stir zone which suffers the abnormal grain growth. The average microhardness of the material was about 130–140 HV0.1 which is a typical value for 2519 aluminum alloy after a precipitation-hardening process. Abnormal grain growth in the stir zone and the upper part of thermo-mechanically affected zone seems to have a very low influence on the distribution of the microhardness in the analyzed samples.

**Figure 16:**
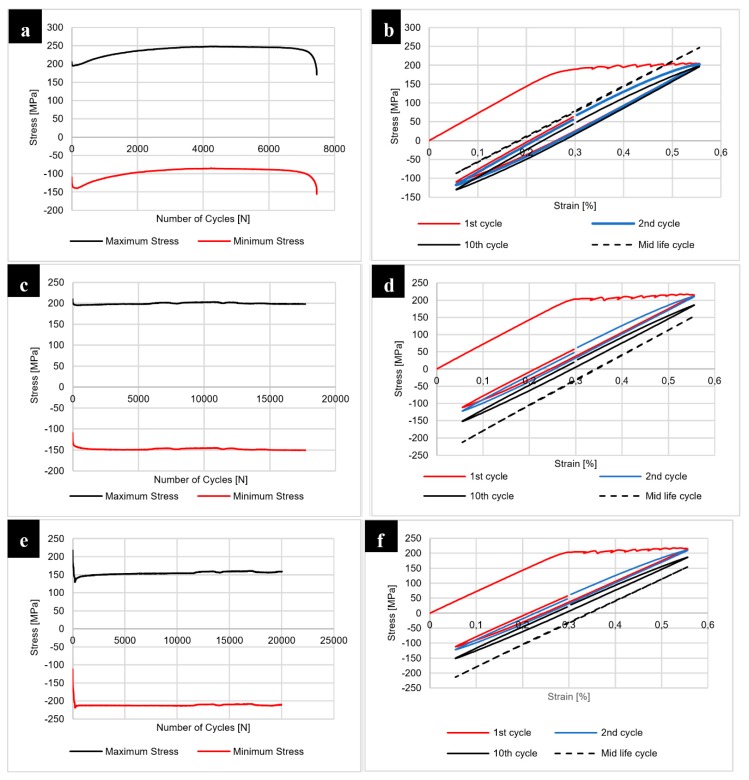
Results of the fatigue testing for 400-100-HT0 sample: (**a**) maximum and minimum stress vs number of cycles, (**b**) hysteresis loop evolution; for 800-100-HT0 sample: (**c**) maximum and minimum stress vs. number of cycles, (**d**) hysteresis loop evolution; for 800-200-HT0 sample: (**e**) maximum and minimum stress vs. number of cycles, (**f**) hysteresis loop evolution.

The results of the fatigue testing of joints in the as-welded state are presented at Figure 16.

**Figure 17:**
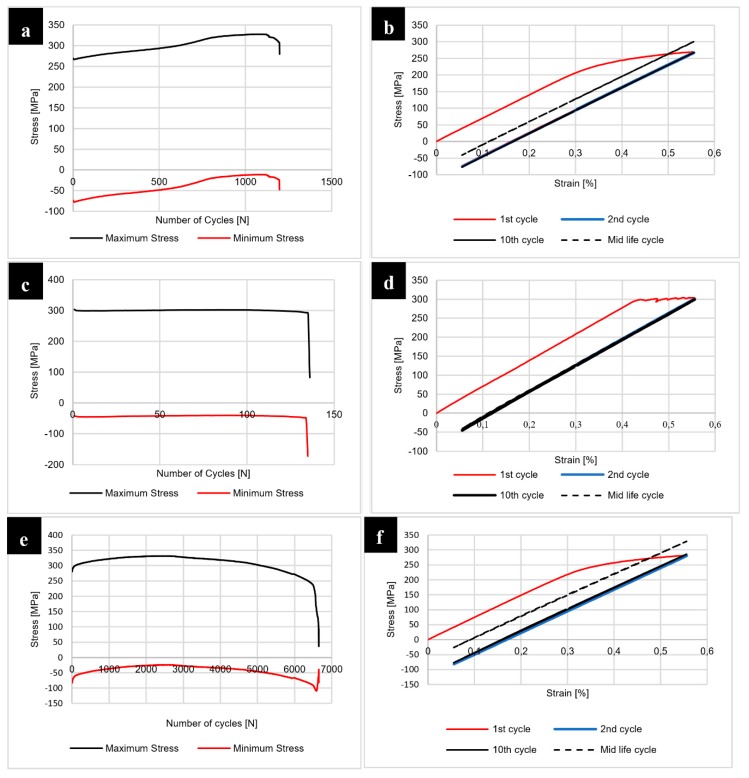
Results of the fatigue testing for 400-100-HT1 sample: (**a**) maximum and minimum stress vs number of cycles, (**b**) hysteresis loop evolution; for 800-100-HT1 sample: (**c**) maximum and minimum stress vs. number of cycles, (**d**) hysteresis loop evolution; for 800-200-HT1 sample: (**e**) maximum and minimum stress vs. number of cycles, (**f**) hysteresis loop evolution.

The fatigue testing of the samples with constant true strain amplitude ε = 0.25% and the cycle asymmetry coefficient equal to R = 0.1 allowed us to investigate the welded joints response to cyclic loading. Our results show that the 400-100-HT0 sample has the lowest fatigue life compared to the joints obtained using the 800 rpm tool rotation speed. The analysis of maximum stress vs. number of cycles curve indicates that in the initial state of testing this joint is characterized by cyclic softening, then it is subjected to the cyclic hardening and the final stabilization (Figure 16a). Although 800-100-HT0 and 800-200-HT0 samples also underwent similar cyclic softening during the initial phase of fatigue testing, they gained their cyclic stability sooner (Figure 16c,e). The analysis of the hysteresis loops evolutions allowed us to state that initial loops have a high participation of the plastic strain (Figure 16b,d,f). The post weld heat treatment influenced the joints response to cyclic loading significantly and the results of the fatigue testing of joints subjected to the post-weld precipitation hardening are presented in Figure 17.

**Figure 18:**
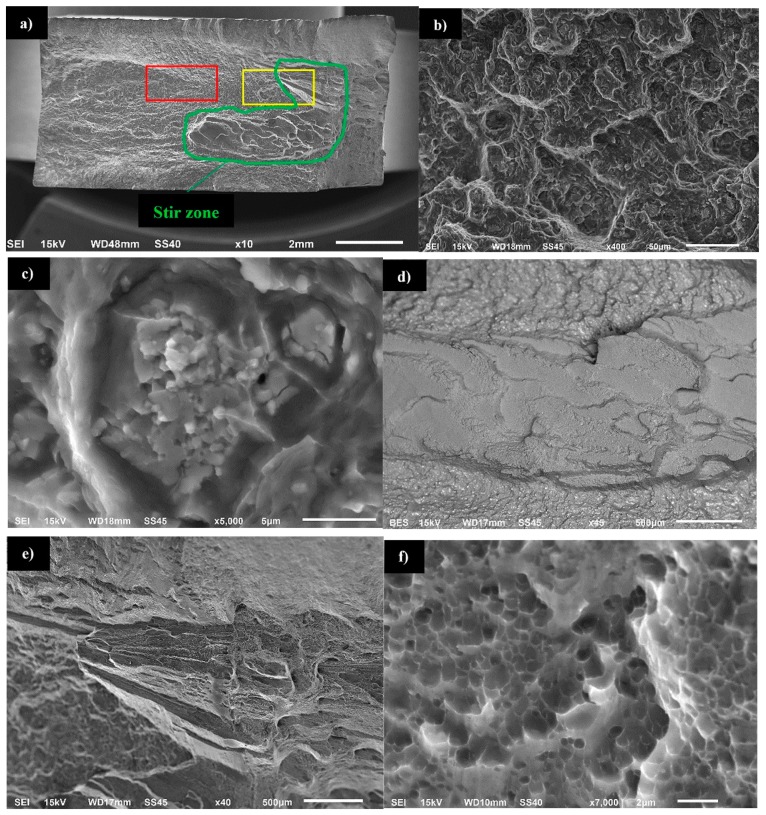
Scanning electron microscopy images of 800-200-HT0 fatigue surface: (**a**) overall view of the fracture surface, (**b**) image of the red marker area, (**c**) magnification of the precipitate, (**d**) back-scattered electron (BSE) image of the stir zone fatigue surface, (**e**) image of yellow area, (**f**) magnification of the fatigue surface in the stir zone.

**Figure 19:**
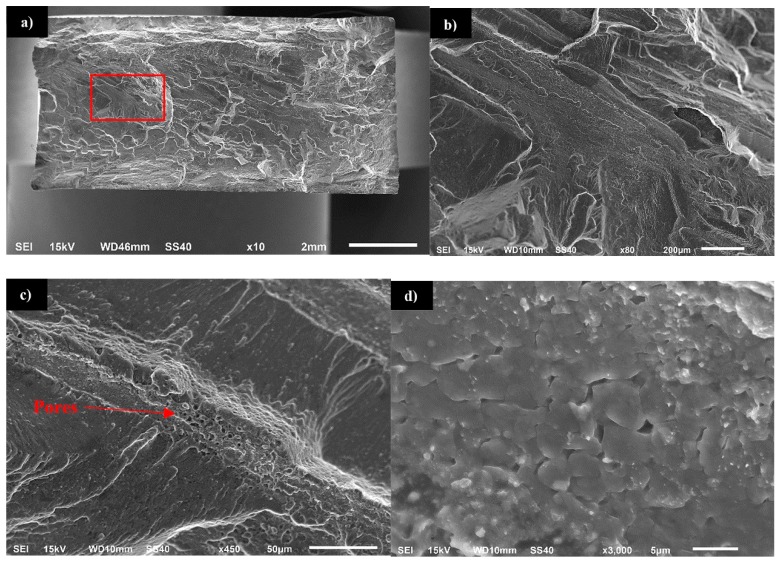
Scanning electron microscopy images of 800-200-HT1 fatigue surface: (**a**) overall view of the fracture surface, (**b**) image of the red marker area, (**c**) band presence in the fracture surface, (**d**) magnification of the band with visible fine grains.

The post-weld heat treatment significantly reduced the fatigue life of all investigated joints. Our results show that samples 400-100-HT1 and 800-200-HT1 in the first phase of fatigue life undergo cyclic hardening (Figure 17a,e). The 800-100-HT1 sample had a cycle stability during fatigue testing but its fatigue life was extremely short (Figure 17c). Decrease in fatigue life of the joints is mostly related to the presence of pores in the structure of welds subjected to the post-weld precipitation hardening process. The analysis of hysteresis loops evolutions confirmed the cyclic hardening of the investigated joints (Figure 17b,d,f). Additionally, the hysteresis loops of the joint subjected to the post-weld heat treatment have a significantly lower participation of plastic strain compared to the joint in the as-welded state (Figure 16b,d,f). The fracture surfaces of the samples 800-200-HT0 and 800-200-HT1 have been subjected to scanning electron microscopy observations (Figure 18 and Figure 19). 

Our results show that the fracture surface of the 800-200-HT0 sample was characterized by mixed ductile and brittle fracture with the predominance of ductile fracture with dimpled texture, especially in the surrounding area of the Al_2_Cu precipitates (Figure 18a,b). The reason of the increasing of plastic deformation participation is that non-coherent, large Al_2_Cu particles caused local stress concentration (Figure 18c). The character of the fracture in the stir zone differs from the rest of the fatigue surface (Figure 18a,e). Additionally, a back-scattered electron (BSE) image of the stir zone fatigue surface allowed to observe that this zone consists very low amount of large precipitates, what confirmed previous scanning electron microscopy observations (Figure 18d and Figure 5a). The magnification of the fatigue surface in the stir zone allowed us to observe that this area is characterized by ultrafine equiaxed dimples (Figure 18f).

Investigation of the fatigue surface of the 800-200-HT1 sample revealed the presence of characteristic bands localized in the center of the examined surface (Figure 19a,b). Compared to the fatigue surface of the joint in the as-welded state, the heat treatment changed the character of fracture to being more brittle and no presence of dimpled texture was observed. Additionally, the occurring of pores in the bands has been revealed (Figure 19c). Further investigation of the bands allowed us to observe a very high participation of intergranular brittle fracture in the center of this area (Figure 19d). The presence of both pores and fine grains in the crack propagating bands suggests that this area was a boundary between thermo-mechanically affected zone and the stir zone, in which after heat treatment the imperfections (pores) were found.

## 4. Discussion

The friction stir-welding technology allowed us to produce Sc-modified AA2519-O joints with no presence of any imperfection in their macrostructure in the as-welded state. The microstructure analysis together with the results of the microhardness distribution on the cross-sections of the samples allowed us to identify typical zones: stir zone, thermo-mechanically affected zone and heat-affected zone. The size of each zone, as well as, their measured microhardness differ depending on the welding parameters used. Our results show that tool rotation speed has a most significant impact on the increase of microhardness in the stir zone, which is characterized by ultrafine grain microstructure with grains sized about 5 μm. Additionally, the results of the chemical composition analysis allowed us to state that in the stir zone Al_2_Cu precipitates undergo the dissolution process due to intense stirring of the welded material in this area. The analysis of the solvus curve in the Al-Cu phase equilibrium diagram allows us to conclude that with the increase of the temperature above 300 °C the solubility of the Al_2_Cu phase into Al alloy also increases significantly [3]. 

Although the dissolution of Al_2_Cu precipitates into aluminum alloy during heat treatment takes time, in the case of the friction stir-welding process the intense mixing of material in the temperature about 400–450 °C promotes the formation of saturated solution in the stir zone. This phenomenon finds its consequences in the fatigue surface analysis. In the case of the zone occurring large Al_2_Cu precipitates (such as base material, heat affected zone and thermo-mechanically affected zone) the fracture is characterized by dimpled texture with precipitates localized in the dimples. Stir zone, which has lack of large precipitates is characterized by ultrafine equiaxed dimples with no visible precipitate presence. This different size of dimples can be explained by the fact that non-coherent, large Al_2_Cu particles cause local stress concentration and as a result the increase of plastic deformation participation in the surroundings of each precipitate. Scanning electron microscopy observations of the stir zone revealed the presence of ultrafine precipitates with about 1 μm size. The results of EDX area analysis of the chemical composition indicate an increased concentration of scandium and zirconium in the precipitates in the stir zone. The analysis of the Al-Sc-Zr phase equilibrium diagram indicates a low solubility of Al_3_(Sc,Zr) precipitates into Al alloy even at the temperature of 600 °C (which exceeded the friction stir-welding process temperature range) [26].

This phenomenon finds its confirmation in similar research concerned with friction stir welding of scandium-modified aluminum alloys [27,28,29]. The Al_3_(Sc,Zr) is the phase coherent with the aluminum matrix and as the results it not causes such stress concentration as non-coherent Al_2_Cu precipitates. For this reason there is not predominant influence of Al_3_(Sc,Zr) precipitates on the dimpled texture of the stir zone in the fatigue surface of 800-200-HT0 sample. The post-weld heat treatment (precipitation hardening) influences the microstructure of joints in a significant way. The grainy structure in the stir zone and in the upper part of thermo-mechanically affected zone suffers abnormal grain growth. This effect of the post-weld heat treatment on the grainy microstructure of high-strength aluminum alloys has been the subject of research [25,30,31,32,33]. The reason of grain overgrowth is a significant thermal instability of grains size mostly in the stir zone. Grains in the stir zone have a large number of dislocations, as well as much distortion energy and on the other hand the energy of their boundary is low due to absence of second phase particles [33]. Abnormal grain growth results in the formation of a very specific boundary between fine, recrystallized grain microstructure and the abnormal grains with the size of millimeters. Additionally, the pores have been found at the boundary between the stir zone and the thermo-mechanically affected zone of the samples in the as-welded state. Despite the imperfections in the joints subjected to the post-weld heat treatment, the results of microhardness distribution analysis indicate a uniform hardening of AA2519 alloy due to precipitation-hardening process with small local fluctuations in microhardness in the abnormal grain growth zone. The changes in the microstructure of heat treated joints found their confirmation in the results of fatigue testing. The post-weld heat treatment significantly reduced the fatigue life of all analyzed samples despite the welding parameters. The observations of the fatigue surface of the samples subjected to heat treatment revealed the crack-propagating bands with the presence of pores and ultra-fine grains. These crack-propagating bands have been identified during microstructure examination as a pore-rich area between large grained and fine grained microstructure.

## 5. Conclusions

Investigation on Sc-modified AA2519 friction stir-welded joint in the as-welded state and after post-weld heat treatment (precipitation hardening) allowed the following conclusions to be drawn:

1. The friction stir-welding process of AA2519-O alloy causes the dissolution of Al_2_Cu precipitates in the stir zone and formation of supersaturated solution in this area;
2. Al_3_(Sc,Zr) precipitates do not dissolve due to the FSW process and form dispersion of fine precipitate in the stir zone;
3. The post-weld heat treatment of obtained joints causes the abnormal grain growth in the stir zone and the upper part of the thermo-mechanically affected zone, as well as the formation of pores in this area;
4. The dissolution of Al_2_Cu in the stir zone finds its consequence in the fracture of the joints. Non-coherent, large Al_2_Cu particles’ presence in the thermo-mechanically affected and heat-affected zone cause local stress concentration and as a result the increase of plastic deformation participation in the surroundings of each precipitate which results in the formation of characteristic dimples on the fatigue surface of the joint. At the same time the stir zone due to the lack of large Al_2_Cu precipitates is characterized by ultrafine dimples;
5. The post-weld heat treatment results in decreasing fatigue life of all analyzed samples. The fatigue surface observations have confirmed that the main reason for this phenomenon is presence of pores and boundary between abnormal and fine grains in the structure of joints investigated.

## Figures and Tables

**Table 1:** Chemical composition of AA2519 alloy to be welded.

Si	Fe	Cu	Mg	Zn	Ti	V	Zr	Sc	Al
0.06	0.08	5.77	0.18	0.01	0.04	0.12	0.2	0.36	Base

**Table 2:** Welding parameters and state for each sample with designation.

Sample Designation	Tool Rotation Speed (rpm)	Tool Traverse Speed (mm/min)	State of the Sample
400-100-HT0	400	100	As-welded
400-100-HT1	400	100	After PWHT
800-100-HT0	800	100	As-welded
800-100-HT1	800	100	After PWHT
800-200-HT0	800	200	As-welded
800-200-HT1	800	200	After PWHT
